# Gene Expression Profiling of Human Vaginal Cells *In Vitro* Discriminates Compounds with Pro-Inflammatory and Mucosa-Altering Properties: Novel Biomarkers for Preclinical Testing of HIV Microbicide Candidates

**DOI:** 10.1371/journal.pone.0128557

**Published:** 2015-06-08

**Authors:** Irina A. Zalenskaya, Theresa Joseph, Jasmin Bavarva, Nazita Yousefieh, Suzanne S. Jackson, Titilayo Fashemi, Hidemi S. Yamamoto, Robert Settlage, Raina N. Fichorova, Gustavo F. Doncel

**Affiliations:** 1 CONRAD, Department of Obstetrics and Gynecology, Eastern Virginia Medical School, Norfolk, Virginia, United States of America; 2 Virginia Bioinformatics Institute, Virginia Polytechnic Institute and State University, Blacksburg, Virginia, United States of America; 3 Laboratory of Genital Tract Biology, Department of Obstetrics, Gynecology and Reproductive Biology, Brigham and Women’s Hospital, Harvard Medical School, Boston, Massachusetts, United States of America; Centers for Disease Control and Prevention, UNITED STATES

## Abstract

**Background:**

Inflammation and immune activation of the cervicovaginal mucosa are considered factors that increase susceptibility to HIV infection. Therefore, it is essential to screen candidate anti-HIV microbicides for potential mucosal immunomodulatory/inflammatory effects prior to further clinical development. The goal of this study was to develop an *in vitro* method for preclinical evaluation of the inflammatory potential of new candidate microbicides using a microarray gene expression profiling strategy.

**Methods:**

To this end, we compared transcriptomes of human vaginal cells (Vk2/E6E7) treated with well-characterized pro-inflammatory (PIC) and non-inflammatory (NIC) compounds. PICs included compounds with different mechanisms of action. Gene expression was analyzed using Affymetrix U133 Plus 2 arrays. Data processing was performed using GeneSpring 11.5 (Agilent Technologies, Santa Clara, CA).

**Results:**

Microarraray comparative analysis allowed us to generate a panel of 20 genes that were consistently deregulated by PICs compared to NICs, thus distinguishing between these two groups. Functional analysis mapped 14 of these genes to immune and inflammatory responses. This was confirmed by the fact that PICs induced NFkB pathway activation in Vk2 cells. By testing microbicide candidates previously characterized in clinical trials we demonstrated that the selected PIC-associated genes properly identified compounds with mucosa-altering effects. The discriminatory power of these genes was further demonstrated after culturing vaginal cells with vaginal bacteria. *Prevotella bivia*, prevalent bacteria in the disturbed microbiota of bacterial vaginosis, induced strong upregulation of seven selected PIC-associated genes, while a commensal *Lactobacillus gasseri* associated to vaginal health did not cause any changes.

**Conclusions:**

*In vitro* evaluation of the immunoinflammatory potential of microbicides using the PIC-associated genes defined in this study could help in the initial screening of candidates prior to entering clinical trials. Additional characterization of these genes can provide further insight into the cervicovaginal immunoinflammatory and mucosal-altering processes that facilitate or limit HIV transmission with implications for the design of prevention strategies.

## Introduction

According to the latest global estimates from UNAIDS, there were about 35 million people living with HIV in 2013 (UNAIDS. GAP report; 2014, www.unaids.org). During the same time, there were around 2.1 million new HIV infections, and 1.5 million people died of HIV-related causes. Women represent slightly more than half of the infected population worldwide and approximately 60% in sub-Saharan Africa, where gender inequalities increase women’s vulnerability to HIV. In some regions young women and adolescent girls account for a disproportionate number of new infections among young people and those living with HIV (UNAIDS. GAP report, 2014, www.unaids.org).

The main route of male-to-female HIV transmission is through the epithelium of the female genital tract exposed to virus-containing semen [1]. Vaginal topical antiviral microbicides represent a novel female-controlled method of prevention of sexual transmission of HIV. During the past 20 years, numerous microbicide products have been developed, with a handful of them reaching advanced clinical trials [2–5]. Thus far, only the nucleotide reverse transcriptase inhibitor (NRTI), tenofovir, tested in the CAPRISA 004 study demonstrated a moderate reduction in HIV acquisition [6]. All other candidate microbicides were ineffective in preventing primary HIV infection. Moreover, one of the first candidate microbicides, a non-ionic detergent nonoxynol-9 (N-9), increased the risk of HIV infection among frequent users. It was later demonstrated that this compound altered epithelial integrity and induced a mucosal immunoinflammatory response leading to recruitment of immune cells, which are potential targets for HIV infection [7]. Two other microbicide candidates, C31G (Savvy) and cellulose sulfate (CS) which failed in phase III clinical trials also proved to alter the mucosal microenvironment [8,9].

Currently, there are multiple candidate microbicides in preclinical development and a handful of products undergoing clinical studies [3,10]. The new candidates have diverse modes of action, including inhibition of HIV attachment, fusion and entry, replication, integration, and other less defined mechanisms. Preclinical studies of products with multiple mechanisms of action are also in progress [3,10]. Lessons learned from the failures of the first generation microbicides underscore the need for early testing of candidate microbicides for their possible adverse effects on the cervicovaginal and rectal mucosae.

The healthy female genital epithelium presents a strong barrier to viral invasion. HIV transmission is estimated to be 1–2 cases per 1000 coital acts [11,12]. However, affected by complex host and viral factors, transmission rates can be much higher [11]. Increased HIV-1 cervicovaginal transmission is strongly associated with inflammation and general immune activation of the cervicovaginal mucosa—conditions that stimulate influx of HIV target immune cells to the mucosal surface, increase the receptivity of these cells to HIV-1, and are often accompanied by epithelial lesions, which facilitate HIV-1 access to its target cells [13–24]. To avoid these adverse effects, the proinflammatory potential of microbicide candidates should be evaluated early during their primary screening [25,26]. Currently, cell-based, explant-based and animal-based models are used to define and preclinically assess potential biomarkers of inflammation, which include cytokines, chemokines and some other molecules [25,27–32]. Biomarker selection in these assays relies on prior knowledge of their involvement in inflammatory responses.

In this study, we set out to identify novel biomarkers for preclinical *in vitro* assessment of the pro-inflammatory/immunomodulatory potential of microbicides using microarray hybridization technology (gene expression profiling). Our goal was to generate unique gene expression profiles for compounds that were known to cause mucosal inflammatory/immunomodulatory response in order to use these profiles for the characterization of microbicide candidates. To this end, transcriptomes of immortalized human vaginal epithelial (Vk2/E6E7) cells exposed to well-known pro-inflammatory/immunomodulatory compounds (PIC) and non-inflammatory compounds (NIC) were compared. Using microarray technology we identified 20 genes that were consistently deregulated by treatment with PICs but not with NICs, thus presenting a signature specific to compounds with potential pro-inflammatory/ immunomodulatory effects on the cervicovaginal mucosa.

## Materials and Methods

### Materials

TLR ligands, Pam3CSK4 (Pam), imiquimod (IMQ) were purchased from Invivogen (San Diego, CA), macrophage activating lipopeptide 2 (MALP2) was purchased from Alexis Biochemicals (Enzo Life Sciences, Plymouth Meeting, PA). TNF-α was purchased from R&D systems (Minneapolis, MN). N-9 was a gift from OrthoMcNeil Corporation. Other compounds/candidate microbicides were acquired as follows: hydroxyethyl cellulose (HEC)—from Hercules (Hopewell, VA), cellulose sulfate (CS) and dextran sulfate (DS)—from Dextran Products (Scarborough, Ontario), PRO2000—from Indevus Pharmaceuticals (Lexington, MA), tenofovir (TFV)—from Gilead (Foster City, CA), C31G from Biosyn Inc. (Huntington Valley, PA), UC-781—from Regis (Morton Grove, IL), emtricibine (FTC)—gift from Dr. Parang (Keykavous Parang, University of Rhode Island).

Doses of compounds (Table 1) have been defined in our previous studies such that their cytotoxicity, if any, would not exceed 15% [33,34].

**Table 1:** Compounds used for Vk2 cells genome profiling.

Conditions	Treatment	Dose	Number of arrays
Non-inflammatory	HEC	1 mg/ml	9
	CMC	1 mg/ml	9
	culture medium		54
Pro-inflammatory/immunomodulatory			
Cytotoxic surfactant	N-9	12.5 μg/ml	9
TLR ligands (in brackets—corresponding TLRs)	Pam3CSK4 (TLR1/TLR2)	10 μg/ml	6
	MALP2 (TLR2/TLR6)	200 ng/ml	6
	imiquimod (TLR7)	30 μg/ml	6
Pleiotropic pro-inflammatory cytokine	TNF-α	40 ng/ml	6

Doses for candidate microbicides (and placebo) were: for CS, FTC, PRO-2000, TFV, DS, HEC—all 1mg/ml; for UC-781–10 μg/ml, for N-9–12.5 - μg/ml, and for C31G – 6.125 μg/ml.

### Cell culture, treatment with compounds

The vaginal keratinocyte cell line Vk2/E6E7 is a gift from Dr. Fichorova (Brigham and Women’s Hospital [35]. This cell line closely mimics the morphological and functional characteristics of native human vaginal tissue and primary epithelial cells of origin [29,36–41]. Cells were maintained in keratinocyte serum-free medium (Gibco, Invitrogen, Grand Island, NY) supplemented with bovine pituitary extract (50 μg/ml), epidermal growth factor (0.1 ng/ml), penicillin-streptomycin (1%), and CaCl2 (0.4 mM). Cells were grown to ~70–80% confluence and treated for 6 h with agents presented in Table 1. Antibiotics were omitted in bacterial colonization experiments. Cells were collected and RNA extracted for gene expression analysis as described below.

### Bacterial colonization

Bacterial colonization of Vk2/E6E7 cells was performed in the Laboratory of Genital Tract Biology, Brigham and Women’s Hospital (BWH) as described in detail elsewhere [41].

*Lactobacillus* isolate and *Prevotella bivia* were obtained from vaginal swabs and phenotypically characterized by classic microbiology techniques as described before [42]. Because conventional methods of phenotyping cannot identify *Lactobacilli* to the species level, sequencing of the 16S rRNA gene of *Lactobacillus* was performed at the Center for Clinical and Translational Metagenomics at BWH. The genetic analysis classified the *Lactobacillus* isolate as *L*. *gasseri* with 99% identity. Vaginal epithelial cultures (Vk2/E6E7 cells) were colonized with *L*. *gasseri*, representing the Gram-positive facultative anaerobes typical for the normal vaginal flora [43–46], or *Prevotella (P*.*) bivia* representing the anaerobic Gram-negative rods associated with bacterial vaginosis (BV) [47–49].

### RNA isolation

Prior to RNA isolation, Vk2 cells were rinsed three times with cold PBS. Total RNA was then extracted with Trizol (Invitrogen Life Technologies, Carlsbad, CA) and purified using RNeasy mini kit columns from Qiagen Sciences (Qiagen, Valencia, CA) according to the manufacturer’s instructions.

The integrity of total RNA was qualified by Agilent Bioanalyzer 2100 capillary electrophoresis and input amount quantified by Nanodrop ND-1000 Spectrophotometer.

### Microarray expression profiling

Microarray mRNA expression profiling was performed by Asuragen, Inc. (Austin, TX). The mRNA was amplified into cRNA and biotin-labeled using modified MessageAmp-based protocols (Ambion Inc., Austin, TX). Labeled cRNA was fragmented, and hybridized to Affymetrix HG-U133 Plus 2.0 arrays (Affymetrix) according to the standard Affymetrix protocol. The U133 Plus 2.0 chip contains more than 56,000 probesets and includes 38,500 well characterized human genes and expressed sequence tags. Affymetrix raw data were acquired using GeneChip operating software (GCOS 1.3) to yield CEL files.

### Data normalization and statistical analysis

Data were processed and analyzed using GeneSpring 11.5 (Agilent Technologies, Santa Clara, CA). The background subtraction, normalization, and log base 2 transformation of gene signals were carried out using the Robust Multi-array Analysis (RMA) summarization algorithm [50]

For statistical analysis, one-way ANOVA was used for multiple group comparison, followed by multiple testing correction setting the false discovery rate (FDR) at 0.05 using the Benjamini and Hochberg method [51]. Genes/probesets with FDR corrected p-values < 0.05 were considered statistically significant. Next, pair-wise comparisons were performed on probesets having statistical significance to detect how these probesets differ in a treatment condition versus control. Probesets showing fold change differences >2 were considered as differentially expressed. Only those probes that were consistently detected as differential and statistically significant were considered as altered.

### Quantitative real-time PCR (qPCR)

Microarray data for selected genes were validated using quantitative real-time PCR. For the cDNA synthesis 1 μg of total RNA was reverse transcribed using the Reverse Transcription System kit from Promega Corp. (Madison, WI USA). Reverse transcription was primed with Oligo(dT)_15_ in a total volume of 20 μl according to the manufacturer’s protocol.

Quantitative real-time PCR (qPCR) was performed on Roche LightCycler Carousel-based system using 4.05 software. PCR was performed in 20 μl reaction volume containing 1 μl cDNA using LightCycler FastStart DNA Master SYBR Green I (Roche, Indianapolis, IN) according to manufacturer’s instructions. The sequences of the primers used for PCR are presented in S1 Table.

Thermocycler parameters were 95°C for 10 min followed by 45 cycles at 95°C for 10 s, 55°C for 5 s, 72°C for 15 s. Each sample was run in triplicates, and normalized to GAPDH RNA used as the endogenous control. The threshold cycle (C_t_) of GAPDH was used to normalize target gene expression (ΔC_t_). The relative change in gene expression was calculated using the 2^-ΔΔCt^ method [52].

Experiments were performed at least three times, the mean and SD were calculated using Graph Pad software (version 5.01).

### Nuclear and cytoplasmic fractions separation, NFkB activation assay

Vk2/E6E7 cells were exposed to PICs and NICs and harvested within 60 min followed the treatment with 10–15 min interval. Nuclear and cytoplasmic fractions were obtained as described [53] with slight modifications. Cells were rinsed twice with ice-cold PBS, scraped, collected by centrifugation at 1000 x g for 5 min, resuspended in STC buffer (0.25 M sucrose, 10 mM tris-HCl, pH 7.5, 3 mM CaCl_2_) supplemented with protease inhibitor cocktail (BD Bioscience, Bedford, MA). Equal volume of STC buffer, containing 1% Triton X-100 was added to the cell suspension, followed by incubation on ice for 10 min. Nuclear and cytoplasmic fractions were separated by centrifugation at 500 x g for 7 min. Nuclear pellet was resuspended in STC, equal volume of 2 x SDS loading buffer was added to the nuclear and cytoplasmic fractions. Cytoplasmic and nuclear proteins were separated by 10% SDS polyacrylamide gel and transferred to PVDF membrane. IκB-α (degradation in cytoplasm) and NFκB/p 65 (nuclear translocation) were assayed by immunoblotting, as described earlier [34] using corresponding antibodies, both at 1:1000 dilution. Rabbit anti-human polyclonal antibodies, NFκB/p65 and IκB-α, were purchased from Santa Cruz Biotechnology, Inc. (Santa Cruz, CA)

## Results

### Identification of genes discriminating between PIC and NIC by gene profiling of Vk2 cells

To develop biomarkers that may discriminate pro-inflammatory/ immunomodulatory compounds (PICs) from non-inflammatory compounds (NICs) in *in vitro* studies, we employed comparative transcriptional profiling using a microarray technique (Affymetrix U133 Plus 2.0). Well-characterized PICs and NICs (Table 1) were applied to human immortalized vaginal epithelial cells (Vk2/E6E7) that have been selected as an *in vitro* test model. Vk2 cells are similar in characteristics to the cells of the tissue of origin, and have proved to be an adequate model to study vaginal epithelial responses to topical agents [29,35,40]. With the goal of identifying genes that are consistently and significantly changed by PIC as compared to NIC treatments, Vk2 cells were exposed to the following compounds. PICs included a pleiotropic proinflammatory cytokine TNF-α, TLR ligands (TLR-L) such as Pam3CSK4, MALP2, and imiquimod, and the detergent N-9 (for a total of 33 arrays), while NICs were represented by HEC and CMC (18 arrays) as well as growth medium control (GM) (54 arrays) (Table 1). A total of 105 Affymetrix U133 Plus 2.0 arrays were processed and analyzed as described in the Materials and Methods section.

**Fig 1:**
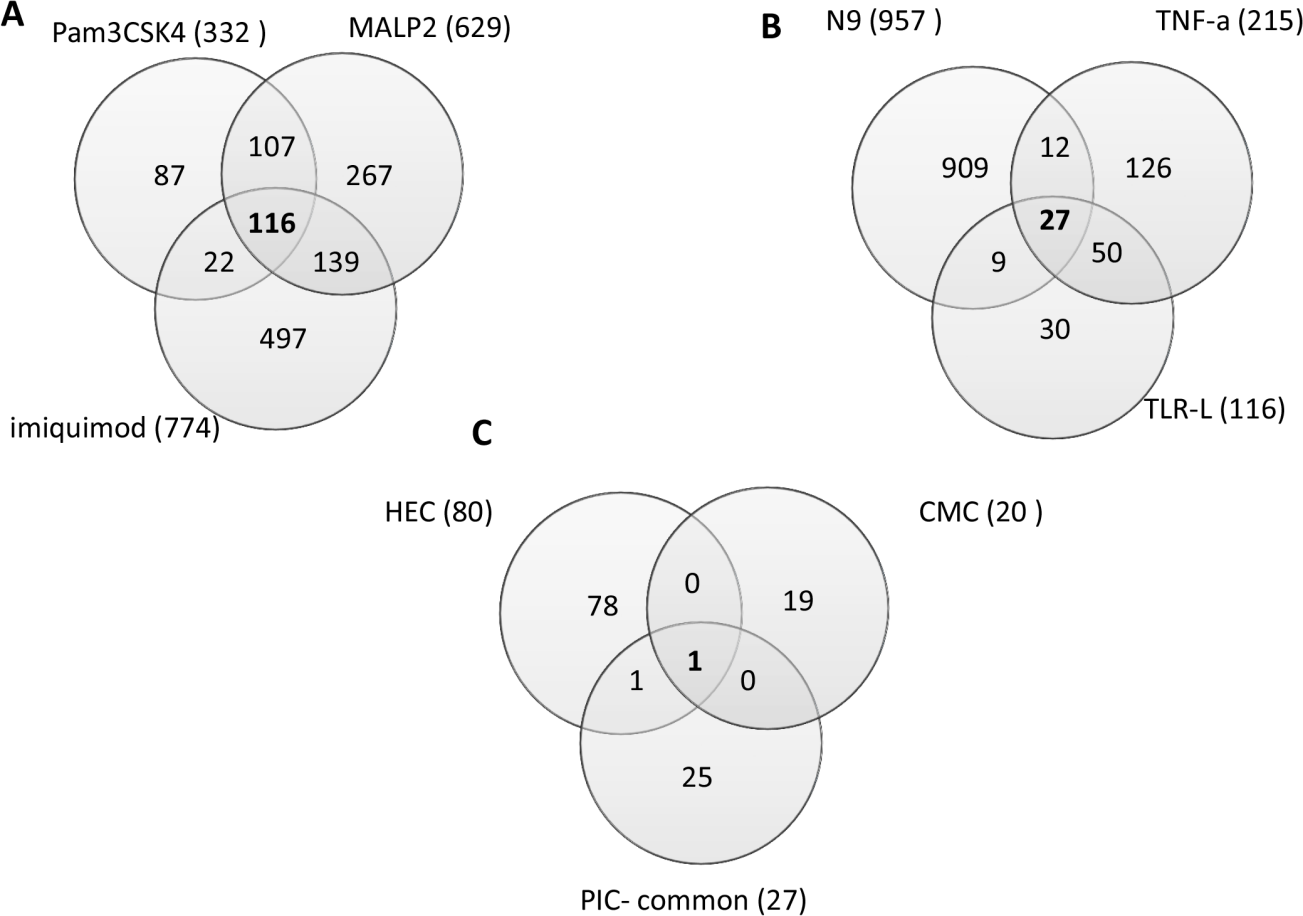
Diagrams showing the number of significantly altered probesets indentified by microarray gene profiling of Vk2 cells exposed to PIC and NIC. Total number of the altered probeserts for each treatment/category is shown in brackets (a gene can be represented by more than one probeset).

The numbers of differentially expressed probesets (fold change cut off = 2, FDR adjusted p-value < 0.05) revealed by microarray analysis in the PIC group were: 215 for TNF-α, 332 for Pam3CSK4, 629 for MALP2, 774 for imiquimod, and 957 for N-9. In the NIC group deregulation was much more modest: 80 probesets for HEC and 20 probesets for CMC (Fig 1, Table 2; S2–S8 Tables).

**Table 2:** Number of differentially expressed probesets in treatment groups compared to control (growth medium).

Treatment	Total	Upregulated	Downregulated
HEC	80	29	51
CMC	20	14	6
TNF-α	215	200	15
Pam3CSK4	332	309	23
MALP2	629	486	143
IMQ	774	493	281
TLR-L shared	116	106	10
N9	957	549	408

Furthermore, the fold changes induced by NICs did not exceed 3.3, while the fold changes in the PIC group reached values >25 for TNF- α, >30 for Pam3CK4, >48 for MALP2, >22 for imiquimod, and >23 for N-9 (S2–S8 Tables).

With the aim of identifying a group of shared deregulated genes, we first analyzed differentially expressed genes within the TLR-L group. There were 116 differentially expressed probesets in common among Pam3CSK4, MALP2 and imiquimod (Fig 1A; S9 Table).

Next, we compared gene expression after all PIC treatments, TNF-a, TLR-L, and N-9. As a result, 27 probesets with significantly altered expression in all PIC treatments were identified (Fig 1B, S10 Table). These 27 probesets encompass 22 genes (some genes are represented by more than one probeset). Next, the expression profile of PICs was compared against that of NICs. Overlap between the PIC-common probesets and probesets differentially expressed by NICs revealed two genes from the PIC list that were also differentially expressed in NICs (Fig 1C, S10 Table). Upregulated by PICs, MMP1 was also upregulated in HEC treatments (fold change 3.3). COL8A1 was downregulted by PICs and both NICs, HEC and CMC (fold change -3.0 and -2.3 respectively). Therefore these genes were excluded from the final list of the PIC-specific probesets. The final list of the probesets altered by PIC but not NIC treatments contains 25 probesets mapped to 20 PIC discriminatory genes (**PIC-DG**) (Table 3, S10 Table).

**Table 3:** Genes differenially expressed in VK2 cells treated with proinflammatory/immunomodulatory compounds.

		Fold change—treatment vs GM					
Gene Symbol	UniGene ID	TNF-α	Pam3CK4	MALP2	imiquimod	N9	Gene name
TNFAIP3	Hs.211600	10.4	5.3	9.4	5.8	3.6	tumor necrosis factor, alpha-induced protein 3
INHBA	Hs.583348	4.8	2.9	5.1	4.8	2.1	inhibin, beta A
G0S2	Hs.432132	4.1	2.8	4.1	4.2	4.3	G0/G1switch 2
CXCL3	Hs.89690	3.7	2.7	8.9	11.5	4.1	chemokine (C-X-C motif) ligand 3
CXCL2	Hs.75765	3.9	5.5	7.9	9.9	4.3	chemokine (C-X-C motif) ligand 2
PRDM1	Hs.436023	3.9	2.3	5.5	6.4	5.9	PR domain containing 1, with ZNF domain
MAFB	Hs.169487	3.3	4.3	5.0	7.1	2.7	v-maf musculoaponeurotic fibrosarcoma oncogene homolog B (avian)
FGF2	Hs.284244	2.2	2.4	2.1	2.5	6.0	fibroblast growth factor 2 (basic)
SERPINB2	Hs.594481	3.1	3.2	3.7	3.6	2.7	serpin peptidase inhibitor, clade B (ovalbumin), member 2
NFKBIZ	Hs.319171	2.4	3.6	4.8	3.1	2.5	nuclear factor of kappa light polypeptide gene enhancer in B-cells inhibitor, zeta
PPP4R4	Hs.259599	2.3	2.5	2.6	2.1	2.2	protein phosphatase 4, regulatory subunit 4
PION	Hs.186649	2.0	2.1	3.0	2.2	2.1	pigeon homolog (Drosophila)
CYLD	Hs.578973	2.4	3.0	2.4	4.6	2.0	cylindromatosis (turban tumor syndrome)
PTGS2	Hs.196384	3.7	3.5	7.5	7.1	12.5	prostaglandin-endoperoxide synthase 2 (prostaglandin G/H synthase and cyclooxygenase)
CCL20	Hs.75498	9.2	19.0	22.2	22.8	3.4	chemokine (C-C motif) ligand 20
OLR1	Hs.412484	25.5	30.4	38.3	22.2	3.9	oxidized low density lipoprotein (lectin-like) receptor 1
SPRR2B	Hs.568239	3.5	9.9	14.4	4.8	2.9	small proline-rich protein 2B
IL8	Hs.624	8.8	16.4	40.2	16.1	4.2	interleukin 8
KRT34	Hs.296942	2.3	3.4	20.0	15.4	2.7	keratin 34
SDPR	Hs.26530	-2.9	-3.1	-4.0	-4.4	-4.8	serum deprivation response (phosphatidylserine binding protein)

**Fig 2:**
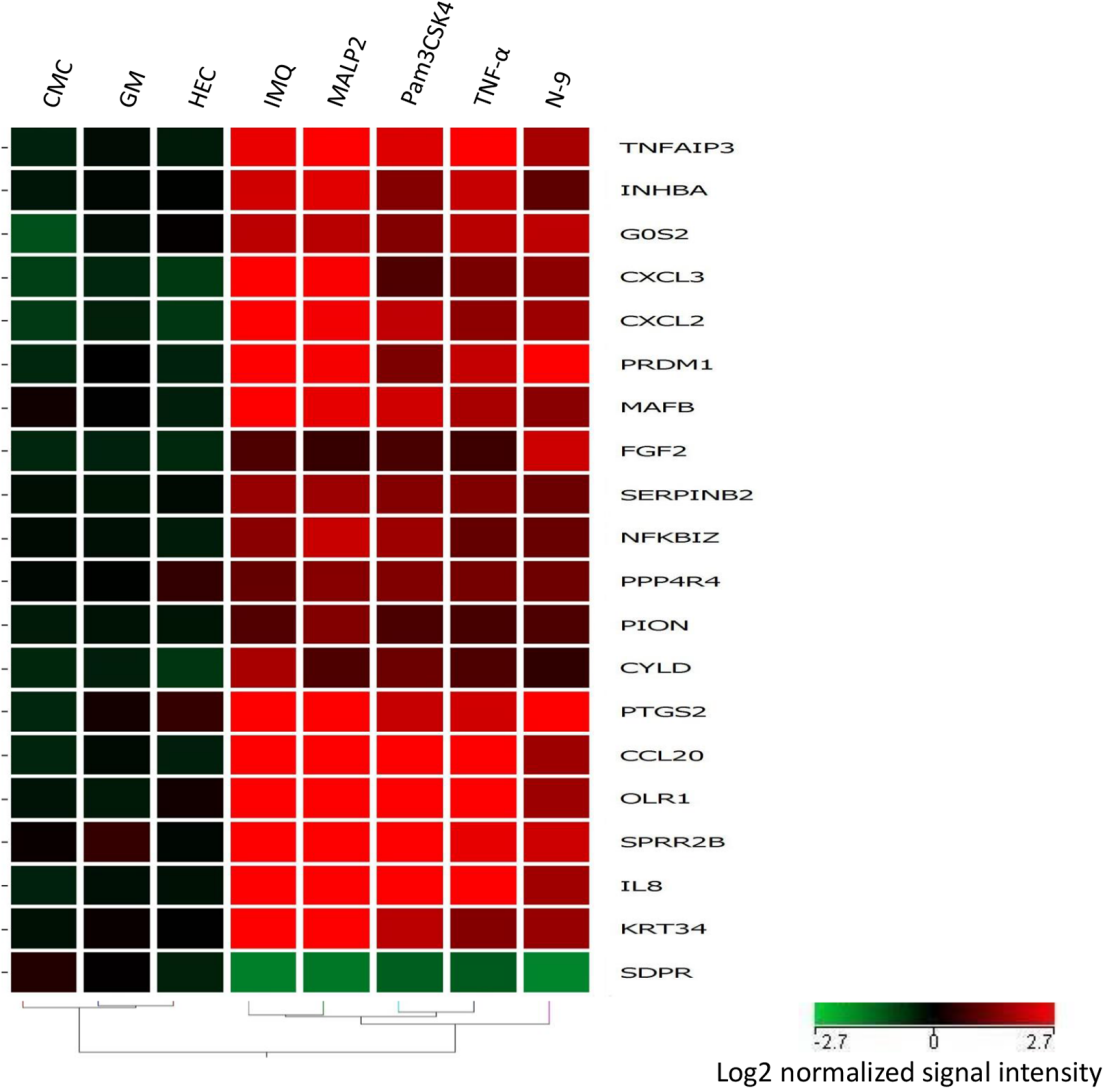
Hierarchical clustering of the 20 discriminatory genes was performed using GeneSpring 11.5 as described in Materials and Methods. Columns represent treatments, rows represent genes. Gene expression levels are indicated by color: red is for upregulation and green is for downregulation. Expression data are averages from at least six experiments/microarrays for each treatment. Hierarchical clustering based on the discriminatory genes demonstrates sharp segregation of the PIC vs NIC treatments.

Of these genes, 19 genes are upregulated, and one is downregulated. A heatmap and hierarchical clustering generated from expression profiles of 20 discriminatory genes common to all PIC treatments of Vk2 cells clearly demonstrates segregation of PICs from NICs (Fig 2).

### Functional analysis of the discriminatory genes

Functional analysis of the PIC-DGs was performed using Ingenuity Pathway Analysis (IPA). All 20 genes were mapped and found to be network-eligible; 18 genes were function/pathway eligible. The top biofunctions for PIC-DGs, as revealed by IPA, are presented in Table 4. Inflammatory response was indicated as the most significant biofunction (p value 3.05E-07–2.05E-0.3), thus supporting pro-inflammatory activity as the principal common feature for the selected PICs, in spite of belonging to diverse molecular and mechanistic categories. Inflammatory/immune modulating response function was attributed by IPA to twelve genes from the PIC-DG list.

**Table 4:** Functional categories of the PIC/NIC discriminatory genes^a^.

Gene	Inflammatory/ immune response	Immune Cell Trafficking	Cellular movement	Cellular Growth & Proliferation	Cell Death	Cancer
P value	4.62E-07 - 3.19E-03	2.24E-07 - 3.19E-03	2.79E-08 - 3.19E-03	6.92E-07 - 3.19E-03	1.17E-06 - 3.19E-03	1.43E-05 - 3.14E-03
PTGS2	x	x	x	x	x	x
CXCL2	x	x	x	x	x	x
CXCL3	x	x	x	x	x	x
FGF2	x	x	x	x	x	x
IL8	x	x	x	x	x	x
INHBA	x	x	x	x	x	x
TNFAIP3	x	x	x	x	x	
NFKBIZ	x	x	x	x	x	
OLR1	x	x	x		x	x
CCL20	x	x	x	x		
CYLD	x			x	x	x
PRDM1	x			x	x	x
MAFB	x		x	x	x	x
SERPINB2	x		x	x	x	x
SPRR2A						x
SDPR						x
G0S2					x	
# Molecules	14	10	12	12	14	13

^**a**^Classification is based on IPA functional analysis and published literature. P values are estimated by IPA

We extended the IPA-generated list of genes belonging to this category by including PIC-upregulated *MAFB* and *SERPINB2* (see below and Discussion) based on recently published studies indicating their link to the inflammatory/ immunomodulatory processes [54,55]. Four genes in the category of inflammatory/immune response, *CCL20*, *IL8*, *CXCL2*, *CXCL3*, encode for chemokines that are involved in immune cells trafficking. IL8 is also known as one of the major mediators of inflammatory responses. Other genes in this category include *OLR1*, *PTGS2*, *TNFAIP3*, *CYLD* and *NFKBIZ*. OLR1 is a receptor for oxidatively modified low density lipoprotein (oxLDL) that upon binding by its ligand induces activation of NFκB (nuclear factor kappa B), the master complex in immunoinflammatory response. PTGS2 (prostaglandin synthase-2) also called COX-2 (cyclooxygenase-2) is one of the key enzymes involved in inflammatory processes [56,57]. TNFAIP3 (A20) and CYLD are deubiquitinating enzymes that play a prominent role in inflammatory signaling by regulating NFkB activation. Another molecule that is tightly associated with NFκB regulation is NFKBIZ, NFκB inhibitor-zeta, also known as I kappa B zeta.

Other important top biofunctions/diseases include immune cell trafficking, cellular movement, cellular growth and proliferation, cancer, and cell death (Table 4).

**Fig 3:**
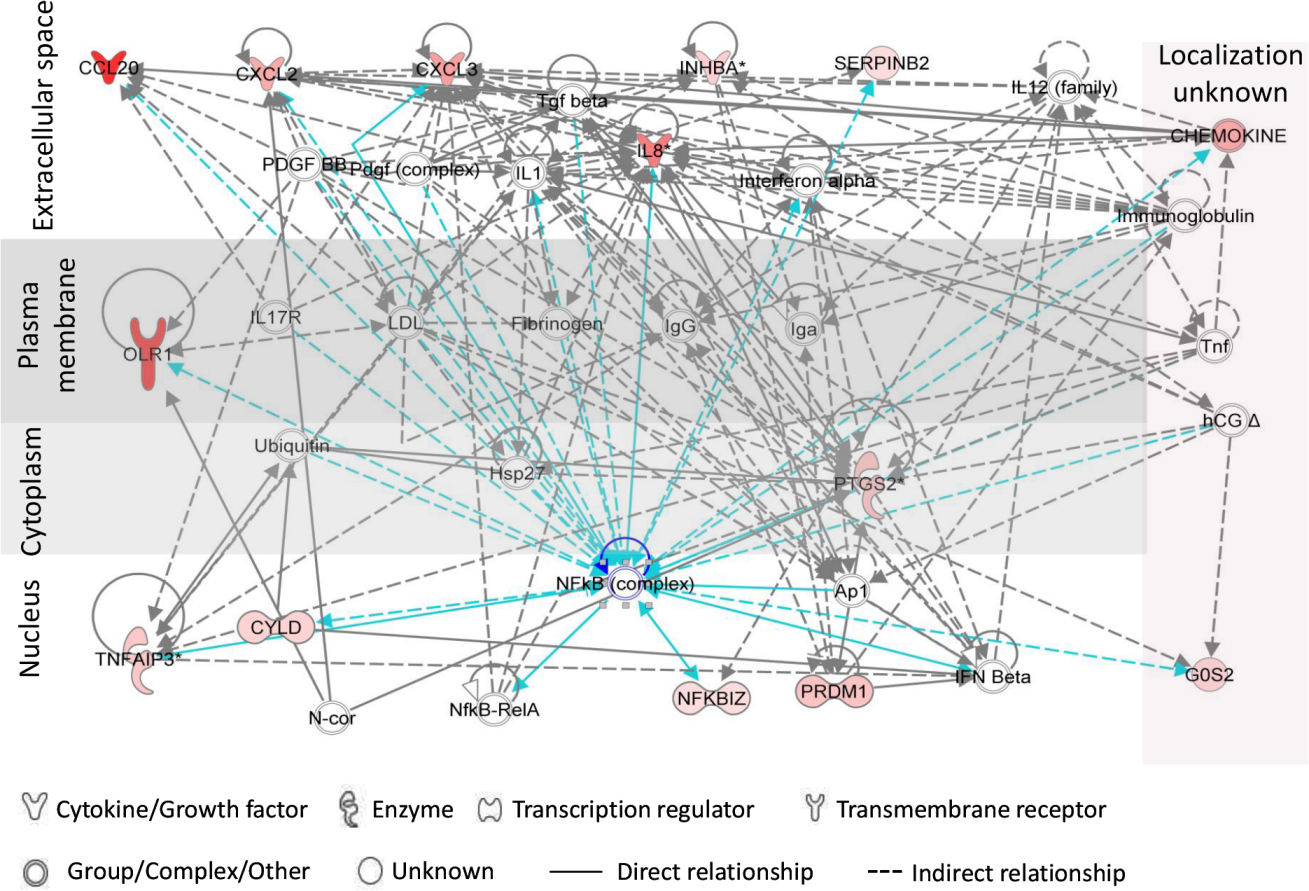
Top network of Vk2 PIC/NIC discriminatory genes generated by Ingenuity Pathway Analysis. Red/pink color indicates upregulation of the genes (microarray data). Connections of NFkB complex with other genes is shown in blue color.

Functional analysis (by IPA) of relationships between PIC-DGs highlighted inflammatory response and cellular movement as the highest scored hypothetical gene networks (Fig 3).

**Fig 4:**
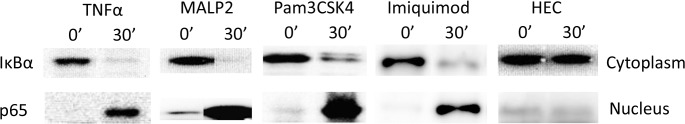
NFκB activation in Vk2 cells in response to PIC treatments. Fast degradation of IκB-α in the cytoplasm and translocation of p65/NFkB to the nucleus following Vk2 cells exposure to PICs was detected in cytoplasmic and nuclear fractions using corresponding antibodies.

Products of six of these genes are localized to the extracellular space, with functions involved in communication with other cells including chemotaxis of immune cells (Fig 3). Of importance, a factor assigned by IPA as a central mediator for most of the PIC-DGs is nuclear factor kB (NFκB), which is a key molecule in the control of inflammatory and immune responses [58–62].We have earlier demonstrated that N-9 activates NFκB signaling pathway in Vk2 cells [34]. In the present study we validate the IPA-identified central position of NFκB in gene deregulation induced by TNF-α and TLR ligands in human vaginal cells. We demonstrate that following the treatments, fast degradation of IκB-α takes place in the cytoplasm accompanied by release and translocation of p65/NFκB to the nucleus indicating NFkB activation (Fig 4).

### Real-time qPCR analysis of selected genes

**Fig 5:**
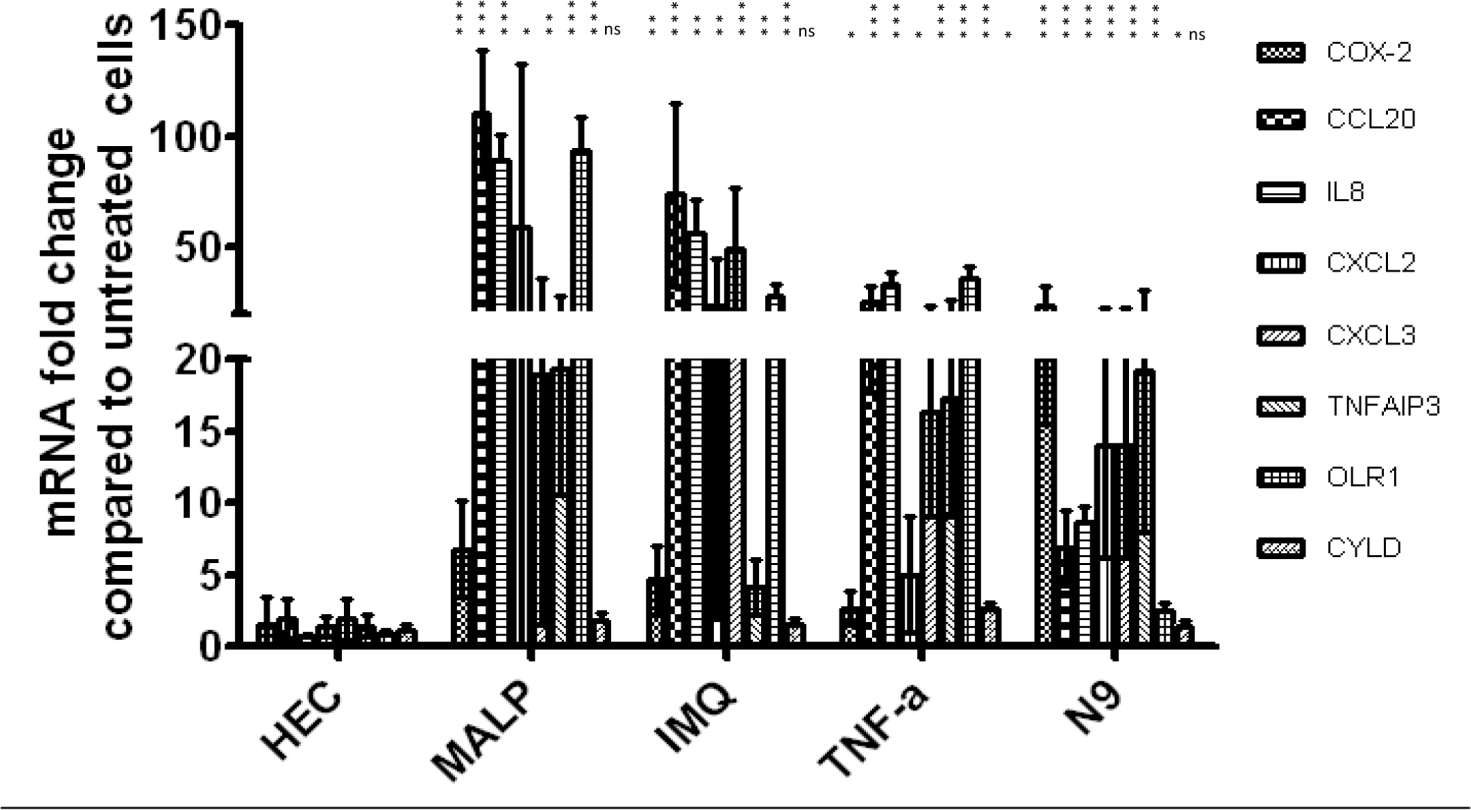
Real-time qPCR validation of changes in expression of eight selected genes observed in microarray analysis. Bars represent the mean ± SD of fold change relative to growth medium control. At least 3 independent experiments were performed for each treatment. All genes were normalized to GAPDH. Asterisks placed vertically denote p values for each PIC treatment relative to HEC used as a reference. (***p<0.0005, **p<0.005, *p< 0.05; Student t-test)

Based on their functions and level of expression, eight genes, *PTGS2*, *CCL20*, *IL8*, *CXCL2*, *CXCL3*, *TNFAIP3*, *OLR1* and *CYLD*, were selected for validation by qPCR. All selected genes, except for *CYLD* showed significant upregulation (Fig 5), thus confirming the microarray data.

Generally, most of the genes tested demonstrated higher fold changes with qPCR compared to microarray. A similar trend was also described by others [63,64].

### Evaluation of candidate microbicides using discriminatory genes

**Fig 6:**
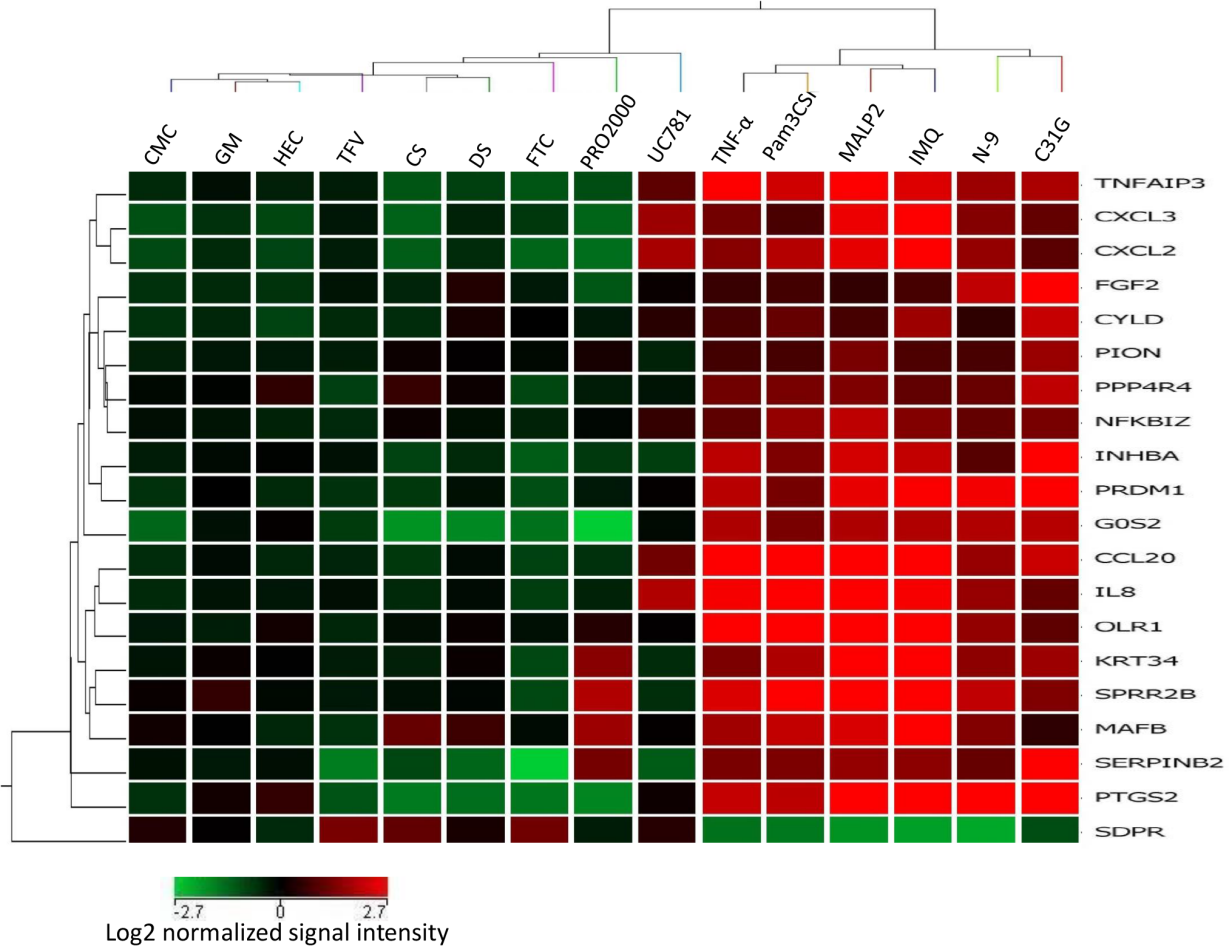
Transcription profile of the 20 discriminatory genes expression in Vk2 cells exposed to candidate microbicides and selected PICs and NICs. Columns represent treatments, rows represent genes. Gene expression levels are indicated by color: red is for upregulation and green is for downregulation. Expression data are averages from at least six experiments/microarrays for each treatment. Clustering based on 20 PIC/NIC discriminatory genes places C31G (known as causing inflammatory response) to the PIC category, while dextran sulfate (DS) and cellulose sulfate (CS)—into the NIC group.

We used a panel of PIC-DG to test their discriminatory power and to evaluate the pro-inflammatory potential of several microbicides /active ingredients: non-ionic detergent C31G (an active ingredient of Savvy), non-ionic polymers—PRO2000, cellulose sulfate (CS), dextran sulfate (DS), reverse transcriptase inhibitors (RTI)—nucleoside/nucleotide RTI (NRTI)—tenofovir (TFV) and emtricitabine (FTC), and non-NRTI (NNRTI)—UC781. The heatmap of cluster analysis of microarray data demonstrates that C31G clusters together with PICs, while the other candidate microbicides proved to be not pro-inflammatory by this gene expression profile (Fig 6).

**Fig 7:**
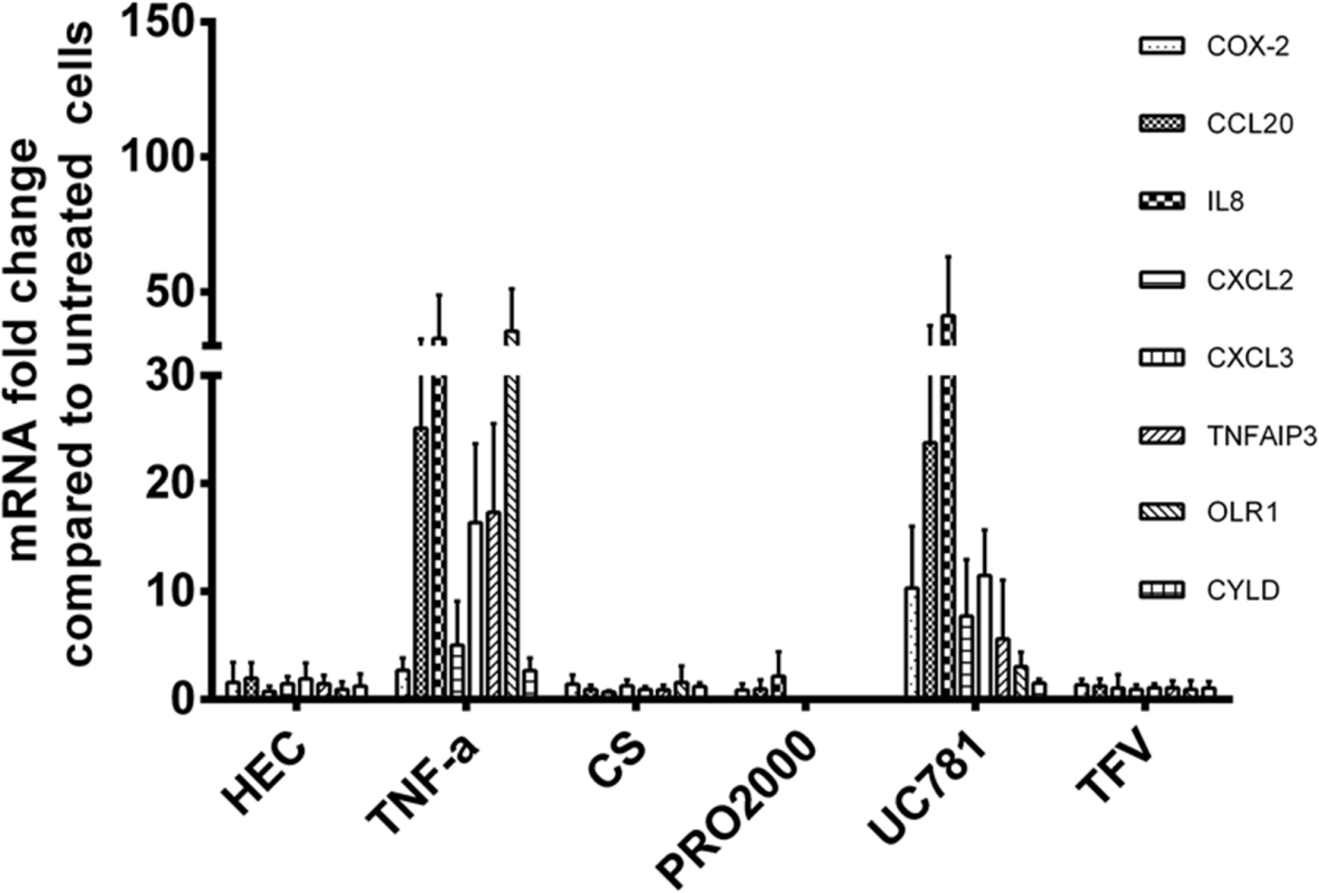
Real-time qPCR validation of changes in expression of eight selected genes observed in the microarray analysis of microbicide candidates. Experimental details are as in Fig 5. HEC and TNF-α are added as references.

It should be noted, however, that although UC781 clustered with the NIC group, it caused upregulation of several of the PIC-DGs belonging to inflammation/immune response category which was confirmed by real-time RT-PCR analysis (Fig 7).

### Panel of selected PIC-DGs clearly discriminates normal vaginal flora- from BV-related bacteria in the Vk2 cell test model

**Fig 8:**
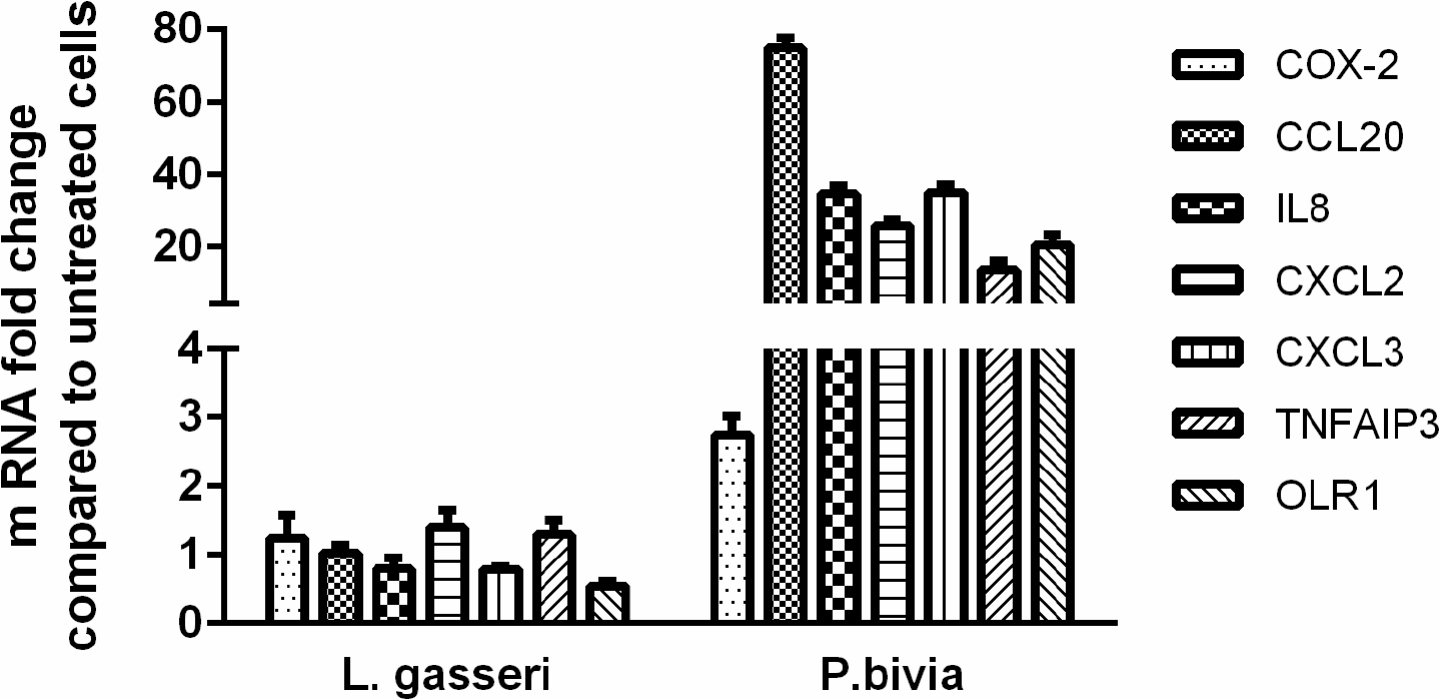
PIC-DG expression following bacterial colonization of Vk2 cells as revealed by quantitative real time RT-PCR. P. bivia (right) induced strong upregulation of all seven PIC-DEGs, while L. gasseri (left) did not cause any changes. Results are presented as mean ±SD of three experiments.

Earlier we demonstrated that *P*.*bivia*, bacteria associated with BV caused significant NFkB activation and increase in IL-8 level in Vk2 cells [41]. In the study reported herein, vaginal epithelial expression of seven selected PIC-DGs, *PTGS2*, *CCL20*, *IL8*, *CXCL2*, *CXCL3*, *TNFAIP3*, *OLR1*, following exposure to *P*. *bivia* and beneficial commensal *Lactobacillus (L*.*) gasseri* was compared using qPCR. Strong induction of all seven PIC-DG by *P*. *bivia* (3 to 75 fold increase), but not by *L*. *gasseri*, was observed (Fig 8), thus validating the discriminatory power of these genes in pathologically relevant conditions of the cervicovaginal tract.

## Discussion

The molecular and cellular mechanisms underpinning HIV-1 vaginal transmission are not yet fully understood. Numerous studies indicate that the initial targets of HIV-1 infection could primarily be CD4+ T cells, with dendritic cells (DC), Langerhans cells (LC), and/or macrophages also playing a role (reviewed in [18,65]). Inflammation and immune activation of cervicovaginal mucosa are considered factors that increase susceptibility to HIV infection due to recruitment and activation of HIV-target immune cells and increased production of immunostimulatory factors that paradoxically intensify HIV replication at the sites of viral exposure. [13–18]. In addition, cervicovaginal inflammation may cause disruption of the epithelial barrier, thus providing a portal for viral entry [66,67]. Therefore, it is critical to screen candidate microbicides for potential mucosal immunomodulatory/inflammatory effects. The goal of this study was to develop an *in vitro* method for preclinical evaluation of pro-inflammatory/immunomodulatory potential of new candidate microbicides and identify new biomarkers of mucosal alteration. To this end, we compared gene expression profiles of Vk2 cells treated with PICs and NICs in order to get a panel of genes that are consistently altered by PICs compared to NICs, thus distinguishing between these two groups.

To make the discriminatory genes more encompassing we selected PICs that belong to different molecular and mechanistic groups. TNF-α is a well characterized pleiotropic proiflammatory cytokine [68,69]. N-9, a nonionic cell membrane disrupting surfactant, is a microbicide that increased the probability of HIV-1 transmission in clinical trials, supposedly, due to vaginal mucosa irritation and inflammation [70,71]. Furthermore, cervicovaginal lavages collected from women who had used N-9 for three days enhanced HIV replication *in vitro* [40]. The other pro-inflammatory group included different TLR-ligands (TLR-L) (Table 1). TLRs are signaling transmembrane proteins that recognize and rapidly respond to various conserved microbial pathogen-associated molecular patterns (PAMPs). The interaction of TLRs with their corresponding PAMPs leads to initiation of innate immune response and triggers pro-inflammatory pathways, in which activation of NFκB plays a central role (see reviews [58,59,61,62]. There are 10 TLRs identified in the human thus far. The female lower genital tract mucosa was found to express most of them [37,72–75]. There is strong evidence supporting the link between the presence of infectious conditions in the lower genital tract, including sexually transmitted infections (STI) and BV, and increased risk of HIV-1 acquisition [19–21]. It is possible that stimulation of innate immune and inflammatory signaling by the PAMP-activated cervicovaginal TLRs through cytokine and chemokine induction increases availability of HIV-1 target cells in the mucosal epithelium, facilitating primary HIV-1 infection.

In this study we identified a set of 20 genes of which 19 genes are consistently activated and one gene is downregulated in human vaginal epithelial cells exposed to various compounds that shared proinflammatory/immunomodulatory activity. Our selection of the stimuli was confirmed by the functional analysis of these discriminatory genes using the IPA program and published data, which revealed that out of the 20 selected genes, 14 genes are known to be involved in inflammatory and immune responses. For most of our inflammation-related genes, their role in inflammatory responses is largely understood, albeit primarily in tissues other than the cervicovaginal mucosa. At the same time, novel features that might link them to HIV susceptibility have recently emerged (discussed below).

Although *in vivo* models have obvious limitations compared to the *in vitro* ones, the set of genes or biomarkers reported herein can be tested early in the process of drug discovery or product development to identify compounds with properties that may result in undesirable mucosal effects. In an attempt to validate this set of biomarkers, we tested microbicide candidates previously characterized in clinical studies with known cervicovaginal mucosal effects [2] and we were able to properly qualify those compounds. In clinical trials, CS, PRO2000 and DS appeared to be safe and did not cause adverse mucosal effects/epithelial disruption, while C31G might have caused vaginal alterations comparable to the effect observed with N-9 [2,76–80]. Clustering analysis of the microarray data classified C31G as PIC, while CS, DS, PRO2000 aligned with the NIC group in good correlation with the results from clinical trials. Tenofovir, currently completing a phase III confirmatory trial in the form of 1% vaginal gel, did not show a pro-inflammatory profile. This is in agreement with results from numerous safety studies including the Phase IIb trial, CAPRISA 004 [81–83]. Interestingly, although clustering with NICs, UC781 showed upregulation of certain PIC-associated genes. Product development of UC781 as a vaginal microbicide, which had reached clinical stage, was discontinued due to pharmaceutical and safety issues. High concentrations of UC781 revealed significant changes in the mucosa of macaques and rabbits [84].

In addition, the discriminatory power of seven selected PIC-DGs was confirmed in the bacterial exposure experiment using commensal and pathogenic microorganisms. We found strong induction of these selected PIC-DGs in Vk2 cells by *P*. *bivia* (3 to 75 fold increase), bacteria associated with BV [47–49]. By contrast, *L gasseri*, one of the dominant beneficial *Lactobacillus* species in the vagina of healthy women [43,44,46,85] did not cause any changes (Fig 8). Upregulation of inflammation-related genes by the BV-related bacteria may have pathogenic implications and provide further insight on the mechanisms underpinning increased rates of HIV-1 transmission in women with BV [86,87].

Clearly these new biomarkers do not identify every potential adverse effect on the cervicovaginal mucosa or environment. CS, which failed to protect women from HIV and may have even increased their susceptibility, did not show inflammatory effects, neither in this model nor in clinical studies. However, it has been reported that it might downregulate epithelial junctional proteins or alter the vaginal microbiome facilitating HIV infection [9,88].

In addition to generating a group of new biomarkers of vaginal mucosal alteration, the reported discriminatory genes may also provide clues to better understanding of the mucosal changes that coexist with and may facilitate HIV transmission. For instance, these genes include several widely known chemokines. IL8, is one of the major mediators of the inflammatory response [89,90]. This chemokine, also known as CXCL8, attracts T cells and neutrophils, stimulates adhesion of monocytes to endothelial cells via interaction with its receptors CXCR1 and CXCR2 [91]. High vaginal IL-8 levels were observed at BV [92] and in response to some pro-inflammatory compounds [29]. IL-8 was found to stimulate HIV-1 replication in T cells and macrophages and increase HIV-1 transmission in cervical explants tissues [93,94]. Much less is known about CXCL2 (MIP-2α, GRO-2, GRO-β) and CXCL3 (MIP-2β, GRO-3, GRO-γ) that are structurally related to IL-8. Like IL-8, they also interact with CXCR2 and are involved in inflammatory and immune responses by attracting and activating immune cells [95].

Another chemokine which is significantly upregulated by PICs is *CCL20*, or macrophage inflammatory protein 3α (MIP-3α). CCL20 is the only chemokine that interacts with CCR6 receptor [96] which is expressed by Th17 lymphocytes and by LCs. Several studies indicate that the CD4^+^ Th17 cells are early HIV/SIV preferential targets and are implicated in HIV pathogenesis [97–101]. Recruitment of CCR6-expressing Th17 cells through CCL20-CCR6 interactions was demonstrated in diverse tissues [102–104]. In the vaginal epithelium these interactions may bring more HIV-target cells to the site of viral exposure. CCL20 was shown to be the main chemoattractant for LCs in human vaginal epithelial cells [105]. Besides CCR6, mucosal LCs express HIV major receptors, CD4 and CCR5, and are capable of internalizing virions. It is still debated whether new virions can be produced by LCs and DCs, however, LCs are able to migrate from the mucosa to the lymph nodes where virions can be transmitted to lymphocytes for productive infection. In *in vivo* and *ex vivo* studies, involvement of LCs in HIV sexual infection has been clearly demonstrated [65,106–110]. In *ex vivo* experiments, inflammatory stimuli, TNF-α and Pam3CSK4 (a ligand for TLR1/TLR2 heterodimer) strongly increased HIV-1 transmission by LCs [107]. Conversely, suppression of CCL20 production and possible prevention of LCs and Th17 attraction and activation correlated with restriction of mucosal transmission of SIV, as demonstrated by glycerol monolaureate studies in non-human primates [13].

Upregulation of *PTGS2* by all PICs observed in this study is a strong assertion of the proinflammatory potential of the selected PICs. The protein encoded by *PTGS2*, most often called COX-2 (cyclooxygenase-2), is an inducible enzyme that is expressed in response to various pathophysiological stimuli. It is one of the key enzymes involved in inflammation. It catalyzes the first steps of conversion of arachidonic acid into prostaglandins (PG) that play an important role in inflammatory and immunomodulatory processes [111–113]. One of the major COX-2 products, PGE2 was shown to directly enhance HIV-1 long terminal repeat (LTR) transcription in human T cells [114]. We have earlier demonstrated that PGE2 levels were elevated in the vaginal epithelial cells following COX-2 induction in response to diverse pro-inflammatory/immunomodulatory stimuli [33,34]. Activation of *PTGS2* gene resulting in PGE2 elevation could be a factor contributing to HIV sexual transmission in inflammatory conditions.

*TNFAIP3* (tumor necrosis factor, alpha-induced protein 3) is another gene that plays an important role in regulation of inflammation and immunity and regulates NFκB. We also observed strong upregulation of *OLR1*, a gene for oxidized low density lipoprotein (lectin-like) receptor 1—cell surface protein belonging to the C-type lectin family. *OLR1* can be rapidly activated by a wide range of stimuli including pro-inflammatory and tissue damaging ones. Its activation triggers several signaling pathways including NFκB [115,116].

NFκB signaling pathway is the central regulator of inflammation and immune activation [60]. We demonstrate here that NFκB is activated in vaginal cells in response to all PIC treatments used in this study (Fig 4). The network of PIC discriminatory gene interactions illustrates the central role of NFκB complex in their regulation (Fig 3). Importantly, NFkB binding elements are present in enhancer located in HIV-1 LTR and provide signal-specific activation of HIV expression in response to NFkB stimuli [117,118]. It might be possible that PIC treatments activate NFkB pathway in the TLR-expressing HIV-1-target cells present in the vaginal epithelium. Multiple genes activated by PICs (INHBA, FGF2, PRDM1, MafB, and SERPINB2) are involved in signal transduction or regulation of immune response [54,55,119–121], which can also promote HIV-1 transcription and replication [122].

## Conclusion

Robust comparative gene expression profiling revealed 20 genes (called here PIC-DGs) that were significantly altered by diverse pro-inflammatory/immunomodulatory compounds in human vaginal epithelial cells. Although the pattern of expression of these genes *in vivo* has not been defined yet (a study with this goal is underway), the observed consistent deregulation of these genes by PICs in the vaginal epithelial cell line suggests that PIC-DGs can be employed as *in vitro* molecular markers of potential inflammatory/ immunomodulatory untoward mucosal effects of candidate microbicides and other vaginal products. We believe that *in vitro* evaluation of the immunoinflammatory potential of candidate microbicides using the PIC-DGs defined in this study could help in the initial screening of microbicide candidates prior to entering clinical trials. Further analysis of these genes can provide better insight into the cervicovaginal immunoinflammatory and mucosal altering processes that facilitate or limit HIV transmission having important implications for the design of novel prevention strategies.

## Supporting Information

S1 Table List of primers. (DOCX)

S2 Table Probesets significantly deregulated in Vk2 cells by TNF-α. (XLSX)

S3 Table Probesets significantly deregulated in Vk2 cells by Pam3CSK4. (XLSX)

S4 Table Probesets significantly deregulated in Vk2 cells by MALP2. (XLSX)

S5 Table Probesets significantly deregulated in Vk2 cells by imiquimod. (XLSX)

S6 Table Probesets significantly deregulated in Vk2 cells by N-9. (XLSX)

S7 Table Probesets significantly deregulated in Vk2 cells by HEC. (XLSX)

S8 Table Probesets significantly deregulated in Vk2 cells by CMC (XLSX)

S9 Table Significantly deregulated probesets common to all TLR ligands. (XLSX)

S10 Table Probesets significantly deregulated in Vk2 cells that are common to all treatments with proinflammatory-immunomodulatory compounds. (DOCX)
