# Supplementary material for: Gene Expression Profiling of Human Vaginal Cells In Vitro Discriminates Compounds with Pro-Inflammatory and Mucosa-Altering Properties: Novel Biomarkers for Preclinical Testing of HIV Microbicide Candidates
Source: PLoS One. 2015 Jun 8;10(6):e0128557. doi: 10.1371/journal.pone.0128557 (PMC4459878; doi:10.1371/journal.pone.0128557)
Supplement: S1 Table — (DOCX) [file pone.0128557.s001.docx]

**S1 Table Primers used for qRT-PCR**

| **Gene** | **Sequence** |
| --- | --- |
| IL8 | F5’ – GCCTTCCTGATTTCTGCAGCTC-3’  R5 ’- TGTTGGCGCAGTGTGGTC-3’ |
| PTGS2 | F5’ – TGAGCATCTACGGTTTGCTG-3’  R5’ - TGCTTGTCTGGAACAACTGC-3’ |
| CCL20 | F5’ - GCGCAAATCCAAAACAGACT-3’  R5’ - CAAGTCCAGTGAGGCACAAA-3’ |
| GAPDH | F5’ – GAGTCAACGGATTTGGTCGT-3’  R5’ - GATCTCGCTCCTGGAAGATG-3’ |
| CXCL2 | *SABiosiencePPH00552E; RP 878, BS179 |
| CXCL3 | *SABiosiencePPH00557B; RP908, BS137 |
| TNFAIP3 | *SABiosiencePPH00063A; RP 133, BS 172 |
| OLR1 | *SABiosiencePPHO2319E RP 1949, BS 122 |
| CYLD | *SABiosiencePPH18819E; RP8216, BS 121 |

**Primer sequence proprietary, RP – reference position, BS – band size*
