# Supplementary material for: Gene Expression Profiling of Human Vaginal Cells In Vitro Discriminates Compounds with Pro-Inflammatory and Mucosa-Altering Properties: Novel Biomarkers for Preclinical Testing of HIV Microbicide Candidates
Source: PLoS One. 2015 Jun 8;10(6):e0128557. doi: 10.1371/journal.pone.0128557 (PMC4459878; doi:10.1371/journal.pone.0128557)
Supplement: S10 Table — (DOCX) [file pone.0128557.s010.docx]

| Supplementary Table **10**. **Probesets deregulated in VK2 cells treated with proinflammatory/immunomodulatory compounds** | | | | | | | | |
| --- | --- | --- | --- | --- | --- | --- | --- | --- |
|  |  |  |  | Fold change - treatment vs control (GM) | | | | |
| Affymetrix Probeset ID | UniGene ID | Gene Symbol | Gene name | TNF-α | Pam3CK4 | MALP2 | imiquimod | N-9 |
| 210004_at | Hs.412484 | OLR1 | oxidized low density lipoprotein (lectin-like) receptor 1 | 25.50 | 30.36 | 38.31 | 22.24 | 3.88 |
| 202643_s_at | Hs.211600 | TNFAIP3 | tumor necrosis factor, alpha-induced protein 3 | 10.39 | 5.26 | 9.41 | 5.84 | 3.63 |
| 205476_at | Hs.75498 | CCL20 | chemokine (C-C motif) ligand 20 | 9.24 | 19.02 | 22.23 | 22.83 | 3.38 |
| 202859_x_at | Hs.624 | IL8 | interleukin 8 | 8.76 | 16.42 | 32.78 | 16.07 | 4.21 |
| 211506_s_at | Hs.624 | IL8 | interleukin 8 | 7.32 | 15.44 | 40.22 | 7.40 | 3.62 |
| 202644_s_at | Hs.211600 | TNFAIP3 | tumor necrosis factor, alpha-induced protein 3 | 7.20 | 3.86 | 7.72 | 4.67 | 2.31 |
| 210511_s_at | Hs.583348 | INHBA | inhibin, beta A | 4.81 | 2.86 | 5.06 | 4.84 | 2.09 |
| 204926_at | Hs.583348 | INHBA | inhibin, beta A | 4.29 | 2.73 | 5.15 | 4.56 | 2.05 |
| 213524_s_at | Hs.432132 | G0S2 | G0/G1switch 2 | 4.13 | 2.83 | 4.14 | 4.21 | 4.25 |
| 209774_x_at | Hs.75765 | CXCL2 | chemokine (C-X-C motif) ligand 2 | 3.92 | 5.54 | 7.94 | 9.89 | 4.32 |
| 228964_at | PRDM1 | PRDM1 | PR domain containing 1, with ZNF domain | 3.88 | 2.35 | 5.49 | 6.36 | 5.89 |
| 1554997_a_at | Hs.196384 | PTGS2 | prostaglandin-endoperoxide synthase 2 (prostaglandin G/H synthase and cyclooxygenase) | 3.73 | 3.45 | 7.49 | 7.07 | 12.53 |
| 207850_at | Hs.89690 | CXCL3 | chemokine (C-X-C motif) ligand 3 | 3.67 | 2.70 | 8.91 | 11.55 | 4.15 |
| 204748_at | Hs.196384 | PTGS2 | prostaglandin-endoperoxide synthase 2 (prostaglandin G/H synthase and cyclooxygenase) | 3.64 | 2.97 | 7.30 | 7.15 | 10.73 |
| 208539_x_at | Hs.568239 | SPRR2B | small proline-rich protein 2B | 3.45 | 9.86 | 14.40 | 4.81 | 2.85 |
| 218559_s_at | Hs.169487 | MAFB | v-maf musculoaponeurotic fibrosarcoma oncogene homolog B (avian) | 3.30 | 4.28 | 5.05 | 7.13 | 2.67 |
| 204614_at | Hs.594481 | SERPINB2 | serpin peptidase inhibitor, clade B (ovalbumin), member 2 | 3.10 | 3.16 | 3.75 | 3.57 | 2.67 |
| 221903_s_at | Hs.578973 | CYLD | cylindromatosis (turban tumor syndrome) | 2.45 | 3.05 | 2.41 | 4.57 | 2.01 |
| 223218_s_at | Hs.319171 | NFKBIZ | nuclear factor of kappa light polypeptide gene enhancer in B-cells inhibitor, zeta | 2.39 | 3.61 | 4.84 | 3.12 | 2.48 |
| 233002_at | Hs.259599 | PPP4R4 | protein phosphatase 4, regulatory subunit 4 | 2.34 | 2.48 | 2.61 | 2.07 | 2.20 |
| 206969_at | Hs.296942 | KRT34 | keratin 34 | 2.33 | 3.39 | 20.01 | 15.41 | 2.65 |
| 204421_s_at | Hs.284244 | FGF2 | fibroblast growth factor 2 (basic) | 2.21 | 2.43 | 2.12 | 2.47 | 6.04 |
| 222150_s_at | Hs.186649 | PION | pigeon homolog (Drosophila) | 2.02 | 2.07 | 3.03 | 2.15 | 2.09 |
| 204475_at | Hs.83169 | MMP1 | matrix metallopeptidase 1 (interstitial collagenase) | 2.01 | 2.33 | 3.43 | 12.09 | 12.29 |
| 226237_at | Hs.718575 | COL8A1 | Collagen, type VIII, alpha 1 | -2.12 | -2.22 | -4.15 | -2.33 | -2.14 |
| 222717_at | Hs.26530 | SDPR | serum deprivation response (phosphatidylserine binding protein) | -2.88 | -3.06 | -3.96 | -4.42 | -4.77 |
